# Genetic variation in individuals from a population of the minimalist bacteriophage Merri-merri-uth nyilam marra-natj driving evolution of the virus

**DOI:** 10.1128/mbio.02564-24

**Published:** 2024-10-30

**Authors:** Tze Y. Thung, Alex Hall, Afif P. Jati, Murray E. White, Rebecca S. Bamert, Kher Shing Tan, Cara Press, George Taiaroa, Francesca L. Short, Rhys A. Dunstan, Trevor Lithgow

**Affiliations:** 1 Center to Impact AMR, Monash University, Clayton, Australia; 2 Infection Program, Biomedicine Discovery Institute and Department of Microbiology, Monash University, Clayton, Australia; 3 Department of Microbiology and Immunology, The Peter Doherty Institute, The University of Melbourne, Parkville, Australia; University of Pittsburgh, Pittsburgh, Pennsylvania, USA

**Keywords:** *Klebsiella*, phage receptor, depolymerase, phage baseplate, community module, ethical bioprospecting

## Abstract

In a survey of a waterway on Wurundjeri land, two sub-populations of the bacteriophage Merri-merri-uth nyilam marra-natj (phage MMNM) were isolated on a permissive host, *Klebsiella* B5055 of capsule-type K2, but were distinguished by minor phenotypic differences. The variant phage MMNM(Ala_134_) showed an inhibited activity against *Klebsiella* AJ174-2, and this was used as a basis to select for further variation through experimental evolution. Over the course of an evolution experiment, 20 phages that evolved distinct phenotypes in terms of the morphologies of plaques formed when they infected host *Klebsiella* were subject to whole-genome sequencing. The evolved phages had mutations in a small set of proteins that contribute to the baseplate portion of the phage virion. Phages MMNM and MMNM(Ala_134_) are minimalist phages, with baseplates formed from only five predicted subunits, akin to other minimalist phages Pam3 and XM1. The homology between all three minimalist phages provided a structural framework to interpret the two classes of mutations derived through evolution in the presence of the semi-permissive host: those that affect the interfacial surfaces between baseplate subunits, and those in a base-plate associated tail-fiber. This study evidences that multiple small mutations can be fixed into a sub-population of phage to provide a basis for phenotypic variation that we suggest could ultimately provide for a shift of virus properties, as an alternative evolutionary scenario to the major genetic events that result in more well-studied evolutionary mechanism of phage mosaicism.

**IMPORTANCE:**

Bacteriophages (phages) are viruses that prey on bacteria. This study sampled natural phage populations to test the hypothesis that untapped genetic variation within a population can be the basis for the selection of phages to diversify their host-range. Sampling of a freshwater site revealed two populations of the phage Merri-merri-uth nyilam marra-natj (phage MMNM), differing by a variant residue (Val134Ala) in the baseplate protein MMNM_26. This sequence variation modulated bacterial killing in plaques, and further evolution of the phages on a semi-permissive bacterial host led to a new generation of phages with more diverse phenotypes in killing the bacterium *Klebsiella pneumoniae*.

## INTRODUCTION

Bacteriophages (phages) are viruses that prey on bacteria. Prey specificity varies, with some phages documented to have a broad range across more than one genus of bacteria that they can infect and productively replicate within (1–3). However, most phages have a narrower range of hosts documented for their replication, with many restricted to specific strains from within a single species of bacteria (1, 3, 4). In order to prey on a bacterium, the phage must adsorb to the bacterial cell surface and initiate steps toward disabling the bacterium. In order to truly fulfill the criteria of being a host, the bacterial cell machinery must then drive the replication of new phage virions (5–7). Host killing is limited by whether or not receptors are present to mediate phage binding and the effectiveness of host immune defenses against a given phage. The set of bacterial immune defenses that can inhibit phage replication includes abortive infection (*abi*) mechanisms, in which host cell death is triggered in the early infection events to limit any further replication (8, 9). The initial adsorption of phages onto a bacterial cell surface is often mediated through one or more proteins in the tail structure, and since mutations can alter the specificity of adsorption events (7, 10), host range has been suggested to be a dynamic trait that can expand or contract in response to environmental factors as well as evolution of host surface features that might increase or decrease phage adsorption (4) as well as the immune defenses present in a potential host bacterium (8, 9).

Phage encounters with a bacterium are proposed to be accidental collisions driven by a combination of Brownian motion and the surge of surrounding fluids (11, 12), and the physics of this compound process has now been modeled as differential settling and shear forces in the aqueous milieu (13). Only if the collision of phage and bacterium results in adsorption of the phage can infection of a host be initiated (5–7). In the case of bacteria enveloped in a polysaccharide capsule, such as *Klebsiella pneumoniae*, there is both (i) an increased capture radius due to an extensive capsule extending up to 400 nm from the cell periphery (14, 15), and (ii) potential for a decreased host-range because the adsorption of phages onto the capsular polysaccharide (CPS)—the first step in the host-binding process (16)—is enhanced by the presence of specific protein components in the virion that bind the CPS (7, 17) and can then degrade it enzymatically through hydrolase or lyase activity (18–20). Analysis of the genes in the CPS locus has suggested that *K. pneumoniae* has more than 180 predicted capsule types of distinct carbohydrate chemistry (21, 22). Thus, *Klebsiella* phage either need to engage and infect only one capsule type or carry multiple depolymerase proteins on the phage virion to engage multiple capsule types. Both strategies have been documented: while very many phages are shown to be specific to a single capsule-type, in the phage ΦK64-1, there are 11 genes encoding depolymerases some of which have been acquired from other *Klebsiella* phages by recombination events (23). Through genetic engineering, the grafting of foreign depolymerases to the tails of unrelated phages matches their new host specificity to that of the grafted depolymerase (17, 24–26). Experimental manipulations that are inspired by natural mosaicism derived from simultaneous infection of one bacterial cell by two or more phages and recombination occurring between these phages (27, 28) with the mosaicism emerging as a very common theme in natural phage populations (4, 29–32).

As an alternative evolutionary mechanism, it has been suggested that point mutations could ultimately alter receptor-binding and other phenotypes of phages in ways that would be permissive to altered host interactions as has been documented in the *Bacillus* phage SPO1 and suggested as a potential evolutionary mechanism (4, 8, 33, 34). We have been sampling natural phage populations to test the hypothesis that untapped genetic variation within a population could be the basis for the selection of *Klebsiella* phages to diversify their host-range. The Merri Creek is an old watercourse in a suburban area of Melbourne, and the Wurundjeri Woi wurrung are its Traditional Owners. Recently, the Merri Creek was shown to be home to phages infecting *K. pneumoniae* (35). Here, we show that sampling at a single site on the creek revealed two populations of the phage Merri-merri-uth nyilam marra-natj (phage MMNM), where the virions differ by a variant residue (Val_134_Ala) in the baseplate protein MMNM_26. The two forms, MMNM and MMNM(Ala_134_), leave a characteristic bulls-eye plaque morphology on the hypermucoviscous (capsule type K2) host *Klebsiella* B5055 and display on the virion a depolymerase, MMNM_24, that degrades capsular polysaccharide of the K2-type. Immuno-electron microscopy showed that the depolymerase was attached at the proximal and at the distal ends of the contractile tail-tube of the phage. Taking a community module approach with just two bacterial species and the phage as a “community” (36), short-term experimental evolution experiments were run in the presence of both the permissive host *Klebsiella* B5055 and a semi-permissive host *Klebsiella* AJ174-2. In this scenario, a natural selection favored further variation in tail-located structures that resulted in changes in plaque morphology. This snap-shot of phage evolution in the laboratory suggests that the baseplate structure is of paramount importance in determining host interactions in mixed microbial populations.

## RESULTS

### MMNM and MMNM(Ala_134_) phenotypes

**Fig 1:**
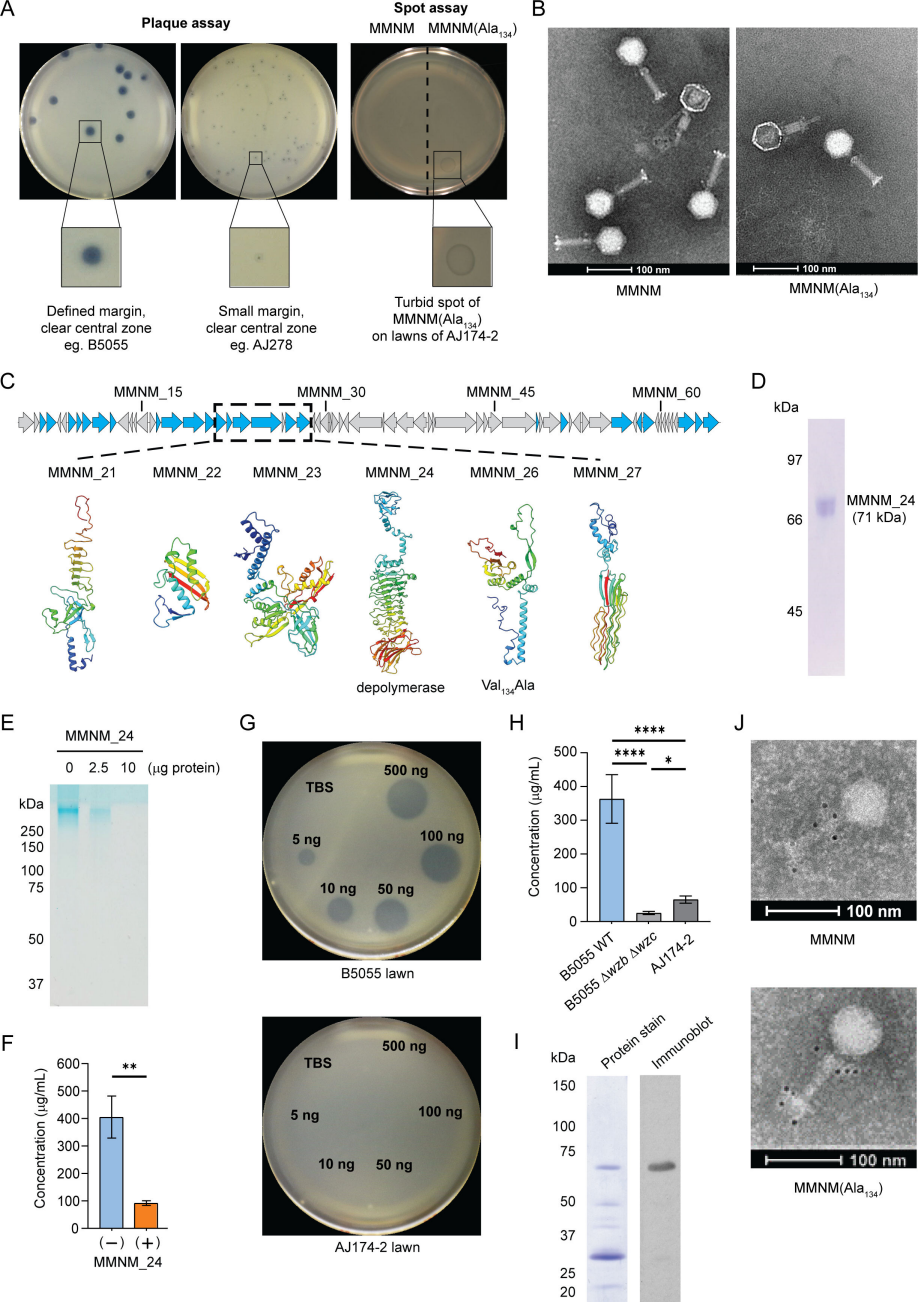
A distinct sub-population of phage MMNM, phage MMNM(Ala_134_). (**A**) Plaques formed on capsule-type K2 strains were scored on lawns of the indicated strains of *Klebsiella* spp. The large bulls-eye plaques with defined margins and clear central zones seen for the isolation host *Klebsiella* B5055 were also seen on *Klebsiella* AJ210, while other strains yielded plaques with small margins and clear central zones such as *Klebsiella* AJ278. Spot-testing of phages on a range of other capsule types showed phage MMNM(Ala_134_) selectively inhibited the growth of *Klebsiella* AJ174-2 and uniquely generated a discernible but turbid spot of clearance on lawns of this strain. (**B**) Negative stain transmission electron microscopy of phages MMNM as previously visualized (35) and MMNM(Ala_134_) virions, each field showing one virion that has contracted. Scale bar is 100 nm. Measurements on micrographs such as these were undertaken, quantifying that the act of contraction leaves the inner tube extended up to 30 nm beyond the outer tube rim (Fig. S1). (**C**) Genome map for phage MMNM highlighting (blue) those structural proteins determined by mass spectrometry of virions (35). Alphafold predictions of protein structures are shown, colored as a rainbow from the N-terminal segments (blue) through to the C-terminal segments (red). The labels “depolymerase” and “Val_134_Ala” are defined in the text. (**D**) Recombinant MMNM_24 was expressed in *Escherichia coli* and purified by affinity and size exclusion chromatography (SEC), visualized by Coomassie blue staining after SDS-PAGE. (**E**) K2 capsular polysaccharide was purified from *Klebsiella* B5055, treated with the indicated amounts of MMNM_24, and then analyzed by 3%–14% SDS-PAGE and staining with Alcian blue. (**F**) Capsular polysaccharide consumption was quantified by the uronic acid method and compared to a sample (+) that had been treated with 1 µg of MMNM_24. Data points are the mean of *n* = 3 biologically independent samples, and the error bars show the SD. (**G**) Spot assays measuring the activity of MMNM_24 using double overlay plates. The top agar was pre-inoculated with *K. pneumoniae* B5055 (capsule type K2) or *K. pneumoniae* AJ174-2 (capsule type K2) before spotting with increasing amounts of purified MMNM_24 (5–500 ng) or a buffer control [Tris-buffered saline (TBS)]. (**H**) Capsular polysaccharide was purified from the indicated three strains and quantified by the uronic acid method. The graphs represent the results from *n* = 3 biological replicates, and the error bars show the SD. Groups were compared using a one-way analysis of variance (ANOVA). (**I**) Phage MMNM virions (10^12^ PFU) were analyzed by SDS-PAGE and Coomassie blue staining to detect all proteins and immunoblotting to detect MMNM_24. The migration position of molecular weight markers is shown. (**J**) Phage MMNM and phage MMNM(Ala_134_) were purified using caesium chloride gradients and subjected to transmission electron microscopy (TEM) analysis. The virions were immunolabeled with antibodies to MMNM_24 and Protein G 5 nm gold conjugate prior to microscopy. Scale bars are 100 nm.

In independent experiments, we identified what were thought to be two distinct *Klebsiella* phages from water samples isolated from the Merri Creek. The phages had been isolated on host *Klebsiella* B5055—which has surface antigens K2:O1, i.e., capsule type K2 and O-antigen type O1—and were then re-tested in spot assays on lawns of various capsule types (16). These two phage preparations behaved differently in the spot assay on a clinical isolate of another antigen type (reported as capsule type K25). However, our further analysis of this clinical isolate, including whole-genome sequencing, revealed that the original respiratory aspirate had yielded two *Klebsiella* strains: one of K25 capsule type forming opaque colonies (genome accession: CP159309–CP159313) and another with a K2 capsule type that formed more translucent colonies (genome accession: CP159314–CP159316). The differential phage sensitivity was observed on the *Klebsiella* (K2:O1) strain. We refer to this strain with the differential phage sensitivity as *Klebsiella* AJ174-2. While neither phage generated observable plaques on *Klebsiella* AJ174-2, one phage generated turbid spots on this strain (Fig. 1A), initially suggesting that they might be different species of phage. For the two phages, the plaque morphology on a range of K2-type Klebsiella strains was similar but not identical (Table S1).

The two phages are indistinguishable via electron microscopy, with an icosahedral head and a tail tube of 92 nm (Fig. 1B). In each case, electron microscopy of the virions revealed that a proportion was in a contracted state (Fig. 1B). These contracted virions have presumably released their DNA, and the tail-tube showed two diameters: a thicker diameter in the capsid-proximal region corresponding to the contracted tail-sheath and a narrower diameter in the distal end of the tail-tube. Clear profiles of the virions (Fig. S1A) were visible from the top end of the tail-sheath to the distal end of the tail-tube (Fig. S1B), enabling measurement of the features in multiple virions (Fig. S1C). This opportunity to measure the length of the tail-tube everted through contraction is important as it provides means to calibrate to the defined dimensions of the bacterial cell envelope (Fig. S1D). The dimensions of the periplasmic space are highly conserved across the Enterobacteriaceae (37) as are the sequences of proteins that span from the inner to outer membrane such as the drug-efflux pump TolC-AcrAB (38) and translocation and assembly module (39, 40). As a result, the structures of these proteins serve as molecular rulers to independently validate direct measurements from electron microscopy (41). In a best-case scenario, where the phage virions infiltrate all the way through the capsule and O-antigen layers to sit as close as possible to the outer surface of the outer membrane, the fully contracted virion might penetrate the peptidoglycan layer but could not reach the inner membrane—let alone penetrate it to enter the cytoplasm of a *Klebsiella* cell (Fig. S1D).

The two phage samples were processed independently for genome sequencing. The genome sequence data showed the two preparations to be individuals of the same species, phage MMNM, belonging to the genus *Jedunavirus* (35). This small phage has a genome size of 47,129 bp, with a GC content of 49.25% and a coding potential for only 67 proteins. Compared to the archetypical contractile phage T4, phage MMNM appears to be constructed on a much simpler blueprint. Mass-spectrometry has defined that there are only 25 structural proteins in the MMNM virion (35) and the tool STEP^3^, an ensemble predictor of phage virion proteins, as well as protein sequence evaluations with HHpred extended the annotations for these protein components (Table 1). The predictions suggest that MMNM virions have a base-plate structure composed of as few as five protein subunits: MMNM_21, MMNM_23, MMNM_24, MMNM_26, and MMNM_27 (Fig. 1C; Table 1). By contrast, the classic phage T4 has a much more complicated baseplate composed of 15 protein subunits (42, 43). For these reasons, we refer to phage Merri-merri-uth nyilam marra-natj as a minimalist phage.

**TABLE 1:** Structural proteins in the virions of phages MMNM and MMNM(Ala134)

ORF	Annotation	Mutations in evolved phages
MMNM_03	Hypothetical protein	
MMNM_04	Hypothetical protein	**+**
MMNM_07	Neck protein	**+**
MMNM_08	Head closure protein	
MMNM_09	Tail-completion protein	
MMNM_10	Hypothetical protein	
MMNM_11	Hypothetical protein	
MMNM_17	Hypothetical protein	
MMNM_18	Tape measure protein	
MMNM_19	Lytic transglycosylase	**++**
MMNM_20	Hypothetical protein	
MMNM_21	Baseplate protein	
MMNM_22	Phospholipase	
MMNM_23	Baseplate J-like protein	**+++++++**
MMNM_24	Tail fiber family protein	**+**
MMNM_26^*a*^	Base-plate wedge protein	**+++++++++++++**
MMNM_27	Putative tail-fiber protein	**+++++++++++**
MMNM_47	HAD superfamily hydrolase	
MMNM_50	Polynucleotide kinase	
MMNM_55	Portal protein	
MMNM_57	Hypothetical protein	
MMNM_58	Head morphogenesis protein	
MMNM_65	Coil containing protein	**+**
MMNM_66	Hypothetical protein	
MMNM_67	Major capsid protein	

^*a*^ The significance of the gray shading indicated that the annotated protein present in all phages (including mutants phages).

Comparison of the genome sequence data showed that the two phage preparations differ only by two indels and one SNP. With numbers referring to the nucleotide position number in the genome, the two indels appear to be due to poly-G phase variations, one of which is in an intergenic region (variant 3958), and the other inducing a frameshift (variant 31679) in hypothetical protein MMNM_45 which is not a component of the phage virion, neither predicted by STEP^3^ nor present in the proteomics analysis of virions (35), and does not have sequence similarity to any proteins of known function. The single SNP (variant 18646) represents a missense substitution in the predicted baseplate wedge protein MMNM_26 (35). This single residue variation, from Val_134_ to Ala_134_, serves as a designation of the two phage virions; hereafter, we refer to the previously described phage MMNM (35) and the new variant as phage MMNM(Ala_134_).

To understand the variation in host-range between phage MMNM and variant phage MMNM(Ala_134_), we sought to address whether the spots formed on *Klebsiella* AJ174-2 lawns were due to a depolymerase with insufficient activity to clear the K2 capsule. Two phage proteins (MMNM_24 and MMNM_27) were both annotated as “tail-fiber protein,” but as with all sequence annotations, these labels need to be taken cautiously (44). Preliminary structural analysis showed that it is MMNM_24 that has the sequence signatures of a depolymerase, including three characteristic domains: an N-terminal domain to tether MMNM_24 to the virion baseplate (colored blue in Fig. 1C), a central β-helical domain containing the enzymatic active site, and a C-terminal domain suggested to function as a non-catalytic carbohydrate binding module (colored red in Fig. 1C).

### The depolymerase on phage MMNM virions hydrolyzes K2 polysaccharide

To determine whether MMNM_24 can hydrolyze K2 capsular polysaccharide, the protein was expressed recombinantly in *E. coli* and purified. On SDS-PAGE, the purified protein migrates according to the predicted molecular size of the monomer at 71 kDa (Fig. 1D), and the purified MMNM_24 catalyzed the degradation of purified capsular polysaccharide as judged by Alcian blue-stained gels (Fig. 1E) and verified by uronic acid assays that indicate the loss of capsular polysaccharide after incubation with purified MMNM_24 (Fig. 1F). In a physiologically relevant demonstration, as little as 5 ng of MMNM_24 was active in clearing a spot in a lawn of *Klebsiella* B5055, yet even 500 ng of the protein failed to clear a spot in a lawn of *Klebsiella* AJ174-2 (Fig. 1G). This result, along with the non-mucoid appearance of the lawn and *Klebsiella* AJ174-2 cell pellets, is consistent with little capsule secretion by *Klebsiella* AJ174-2 (Fig. 1H) but leaves the spots produced (Fig. 1A) by phage treatment of *Klebsiella* AJ174-2 an unanswered issue. The purified MMNM_24 was also used to raise antibodies for immunogold-labeling of phage, imaged by electron microscopy. The monospecific nature of the antibodies was demonstrated by immunoblotting of whole phage particles after SDS-PAGE (Fig. 1I). The immunogold-labeling electron microscopy analysis revealed that MMNM_24 sits in two annuli: around the distal end of the tail-tube, i.e., attached to the base-plate, but also around the collar between the capsid:tail-tube junction (Fig. 1J). Referring to the negative stain profiles of phage MMNM, the location of the depolymerase correlates to the presence of knob-like protrusions at the phage collar and baseplate (Fig. 1B). The MMNM_24 depolymerase is present and similarly localized in the virions of both phage MMNM and variant phage MMNM(Ala_134_).

### TraDIS-based discovery of the phage receptors

**Fig 2:**
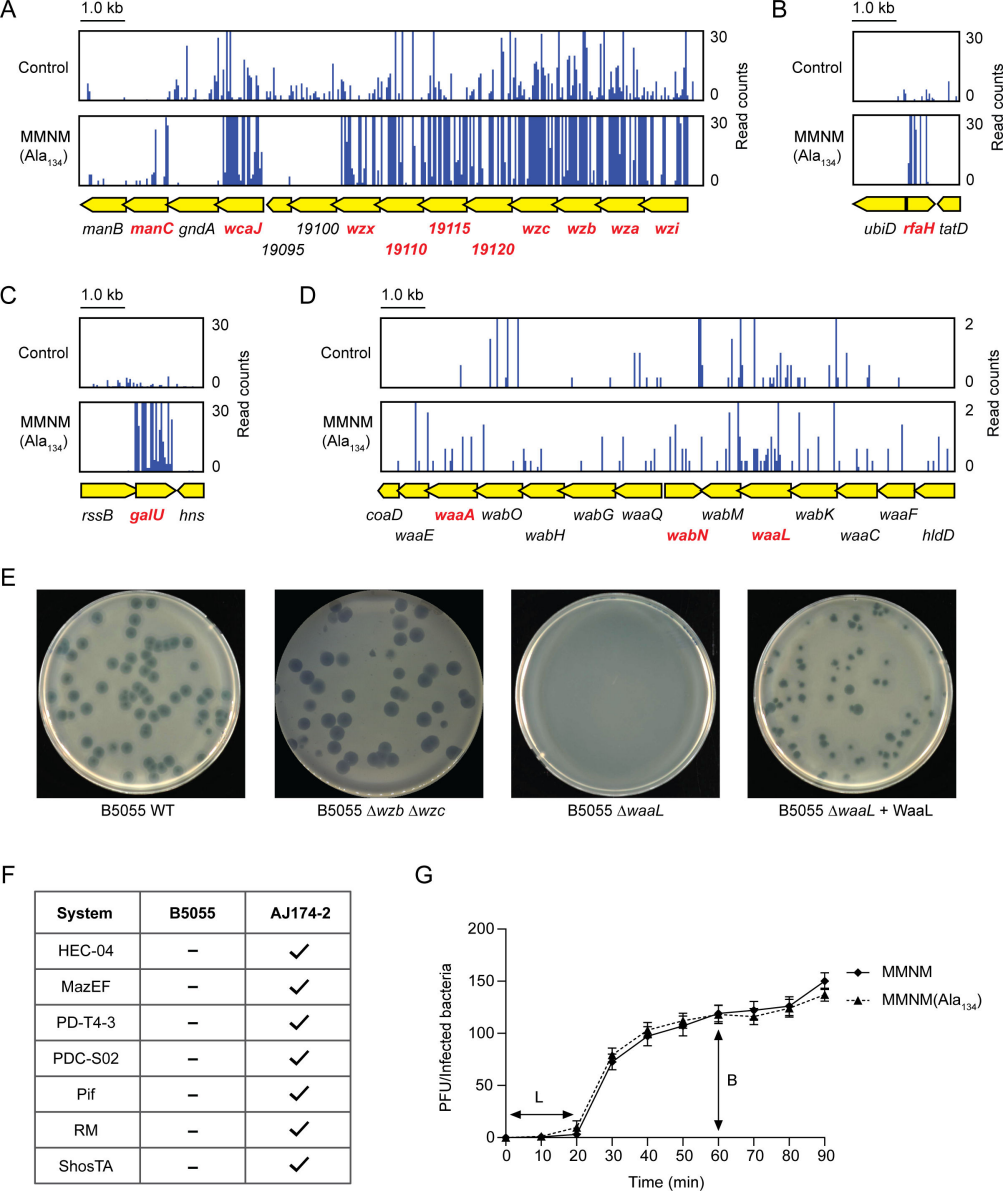
Genetic selection for host genes essential for killing by phage MMNM(Ala134). (**A**) Summary of the TraDIS screen results showing a significant enrichment of transposon insertion sites mapped onto genes (highlighted in red) in the CPS locus. One representative plot from each of the three independent biological replicates is shown. (**B**) Representative plot from each of the three independent biological replicates showing insertions into *rfaH* and (**C**) *galU* that encodes the UDP glucose pyrophosphorylase needed to utilize galactose for CPS synthesis. (**D**) TraDIS screen results showing the transposon insertion sites mapped onto genes (highlighted in red) responsible for O-antigen synthesis. One representative plot from each of the three independent biological replicates is shown. (**E**) Image taken from a representative plaque assay on *Klebsiella* B5055 wild type, B5055Δ*wzb*Δ*wzc*, B5055Δ*waaL*, and the complemented B5055Δ*waaL* mutant expressing plasmid-encoded WaaL. (**F**) Schematic summarizing the predictions from Prokaryotic Antiviral Defence Locator (PADLOC) (47, 48) and DefenseFinder (49) for *Klebsiella* B5055 and *Klebsiella* AJ174-2. (**G**) One-step growth curve of phage MMNM and phage MMNM(Ala_134_) on *Klebsiella* B5055. The latent period “L” and burst size “B” are indicated. Data points are the mean of *n* = 3 biologically independent samples, and the error bars show the SD.

To determine the receptors for phage MMNM(Ala_134_), we screened a highly saturated transposon mutant library constructed in *Klebsiella* B5055 (45) to determine which cell surface feature serves as a receptor for phage binding (Fig. 2A). After growth in the presence of phage, surviving mutants were isolated and sequenced by Transposon Directed Insertion Sequencing (TraDIS) (46). A total of 16 genes were identified which, when disrupted by transposon insertion, conferred survival in the presence of phage MMNM(Ala_134_). Ten of these genes were located in the CPS locus (Fig. 2A), and another, *rfaH*, is a key transcriptional regulator of the CPS locus (Fig. 2B). Transposon insertion into *galU* also resulted in phage resistance (Fig. 2C). Three other genes responsible for lipopolysaccharide biosynthesis*—waaA*, *wabN*, and *waaL*—were also identified in the screen (Fig. 2D).

The anti-terminator RfaH controls both the expression of capsular polysaccharide and O-antigen side chains on the lipopolysaccharide layer of the outer membrane, and GalU is the UDP-glucose pyrophosphorylase that provides UDP-galactose and UDP-glucose as precursors for the biosynthesis of both capsular polysaccharide and lipopolysaccharide. Taken together, the data suggested that both the K2 capsular polysaccharide and the underlying lipopolysaccharide in the cell surface might both function as receptors for phage MMNM. To test this hypothesis and validate the TraDIS data, mutant strains lacking the capsule (B5055Δ*wzb*Δ*wzc*) or O-antigen (B5055Δ*waaL*) were challenged with phage MMNM(Ala_134_), and plaque assays were visualized (Fig. 2E). The phage can form plaques on the B5055Δ*wzb*Δ*wzc* strain, but the morphology of the plaques has crisp boundaries compared to the bulls-eye plaques on the B5055 strain. This suggests that while the phage can degrade capsule when present in order to access the *Klebsiella* cell surface—which is the explanation to bulls-eye plaques—the capsule binding is not a strictly necessary step to productive infection by phage MMNM(Ala_134_). The phage cannot form plaques on the B5055Δ*waaL* strain lacking O-antigen, confirming the lipopolysaccharide as a receptor for phage binding to the *Klebsiella* cell surface (Fig. 2E). Thus, capsule binding is neither necessary or sufficient for infection, as a receptor should be, but O-antigen binding is.

A possible explanation for the positive spot-test data on *Klebsiella* AJ174-2 (Fig. 1A) is that this strain of *Klebsiella* mounts a phage defense, such as an *abi* defense or other defense mechanisms (8, 9), where limited numbers of bacteria die in order to limit phage amplification. DefenseFinder (49) and the Prokaryotic Antiviral Defence Locator (PADLOC) (47, 48) are tools using machine-learning approaches based on hidden Markov model (HMM) profiles for each of the components in the known anti-phage immune systems, though abortive infection systems are difficult to predict (9). Analysis of the *Klebsiella* B5055 and *Klebsiella* AJ174-2 genomes with DefenseFinder and PADLOC indicated a number of putative defense systems present (Table S2) with some of these predicted systems in the *Klebsiella* AJ174-2 strain but not the permissive *Klebsiella* B5055 (Fig. 2F).

Phages MMNM and MMNM(Ala_134_) were isolated on *Klebsiella* strain B5055, and the infection parameters of each phage were defined by the latent period (the time-interval wherein the first round of phage particles are assembled, the host is lysed, and the new virions are released) and the burst size (the total number of progeny virions that are released in this event). One-step growth curves on this strain revealed that phage MMNM(Ala_134_) was indistinguishable from phage MMNM, having a latent period (L) of 20 min and a burst size (B) of 120 (Fig. 2G). Equivalent experiments were conducted using *Klebsiella* AJ174-2 as host and then plating amplified phage on *Klebsiella* B5055 or *Klebsiella* AJ174-2; however, no plaques were observed in any case. Thus, if it is a phage defense process determining the spot clearance of *Klebsiella* AJ174-2, it limits phage numbers to a level below which we can detect in these one-step assays. Which type of phage defense was not explored, but the observation that some—albeit limited—phage MMNM(Ala_134_) could be made in the initial period of phage challenge warranted investigation as this bottleneck could provide a system for identifying point mutations could ultimately provide phage phenotypes in ways that would be permissive to altered host interactions.

### Experimental evolution in the presence of two hosts

**Fig 3:**
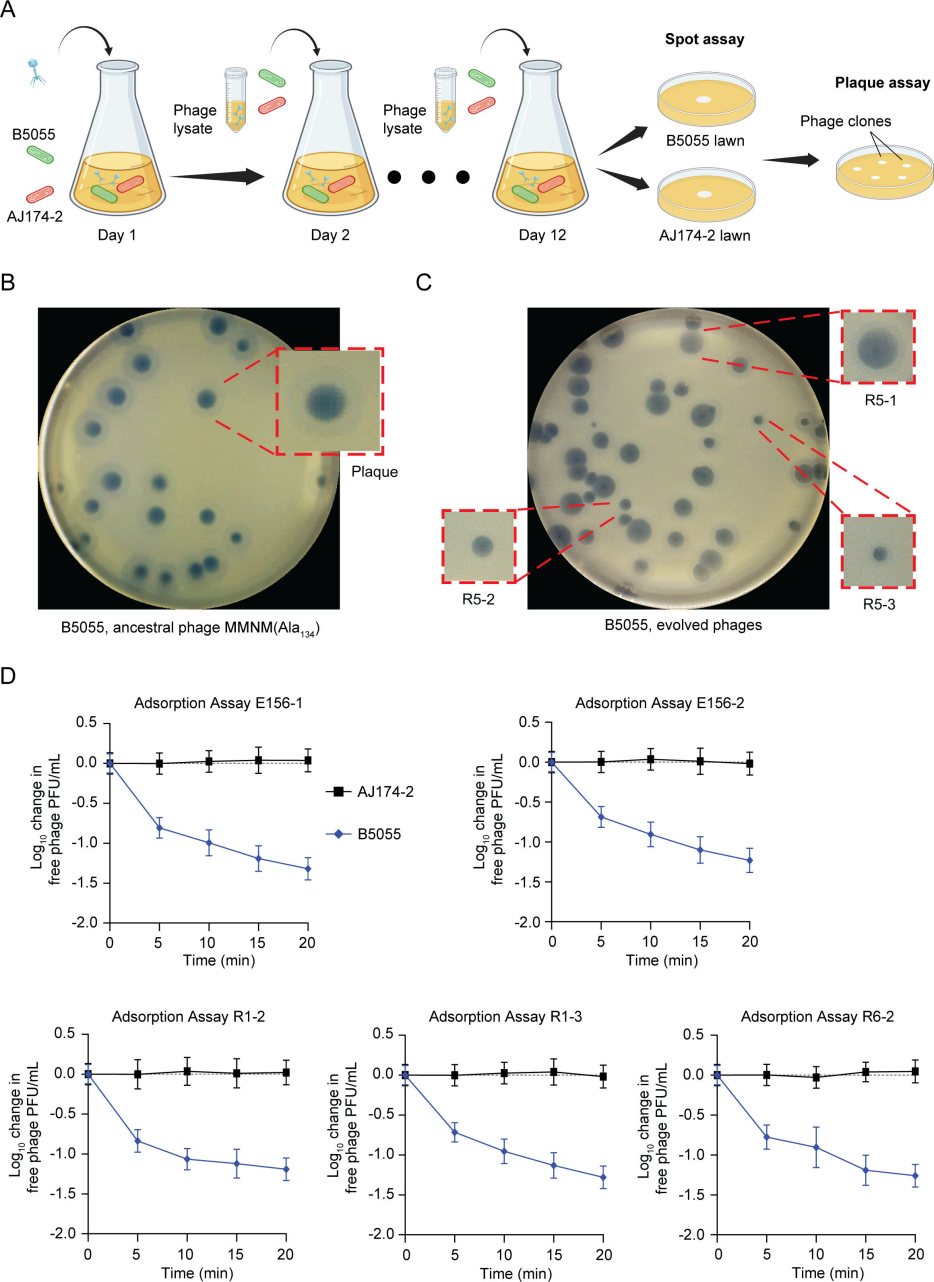
Experimental evolution of phage MMNM toward lytic activity on a new host. (**A**) Schematic summarizing the evolution experiment starting with phage MMNM(Ala_134_) (blue), *Klebsiella* B5055 (green), and *Klebsiella* AJ174-2 (pink). Positive results in the crude spot assay then led to careful evaluation by plaque assays on lawns of *Klebsiella* B5055 (see Fig. S2). Phages isolated and analyzed in this evolution experiment are summarized in Table 2. (**B**) An example of the plaque assays shown for ancestral phage MMNM(Ala_134_) plated on *Klebsiella* B5055. Inset: magnification of a characteristic bulls-eye plaque morphology. (**C**) An example of the plaque assays shown for evolved phage MMNM R5-1, R5-2, and R5-3. Inset: magnification of a characteristic mutant plaque morphologies. (**D**) Phage binding to either AJ174-2 or B5055 was measured by adsorption assays conducted over 20 min. At the indicated time intervals, samples were withdrawn and plated for plaques. Data points are the mean of *n* = 3 biologically independent samples, and the error bars show the SD.

We, therefore, turned to experimental evolution experiments in order to amplify phage MMNM(Ala_134_) progeny that might be detected from the continued presence of the semi-permissive host *Klebsiella* AJ174-2. We established a protocol based on the concept of a community module (36), in this case, consisting of a semi-permissive host to select for phages that had mutated to engage with it for at least a limited amount of replication, and a permissive host to amplify any resultant phages. The simple community of *Klebsiella* AJ174-2 to train phage MMNM(Ala_134_) and *Klebsiella* B5055 to amplify the number of trained phage to detectable levels is represented diagrammatically (Fig. 3A).

**TABLE 2:** Mutations in evolved MMNM phages

Evolved phage	Position	Type	REF^*a*^	ALT^*b*^	Phage protein variant
E156-1^*c*^	12,008	snp	C	T	MMNM_19
	15,895	snp	A	G	MMNM_23
	19,666	snp	T	C	MMNM_27(Val_235_Ala)
E156-2	12,008	snp	C	T	MMNM_19
	15,895	snp	A	G	MMNM_23
	19,733	snp	C	G	MMNM_27(Asn_257_Lys)
R1-1	18,655	snp	C	T	MMNM_26
R1-2	15,895	snp	A	G	MMNM_23
	19,594	snp	C	A	MMNM_27
R1-3	2,921	snp	T	C	MMNM_4
	15,895	snp	A	G	MMNM_23
	19,666	snp	T	C	MMNM_27
R2-1	18,508	snp	T	C	MMNM_26
R2-2	15,895	snp	A	G	MMNM_23
R2-3	18,655	snp	C	T	MMNM_26
	19,645	snp	G	C	MMNM_27
R3-1	3,397	ins	C	CCT	MMNM_6/MMNM_7
	18,655	snp	C	T	MMNM_26
	21,645	snp	A	G	MMNM_33
R3-2	17,956	del	AG	A	MMNM_24
	18,508	snp	T	C	MMNM_26
	19,645	snp	G	C	MMNM_27
R3-3	18,712	snp	A	G	MMNM_26
R4-1	18,508	snp	T	C	MMNM_26
	21,649	snp	C	G	MMNM_33
R4-2	15,895	snp	A	G	MMNM_23
	44,448	snp	G	A	MMNM64/MMNM_65
R4-3	18,625	snp	C	T	MMNM_26
	19,645	snp	G	C	MMNM_27
R5-1	18,508	snp	T	C	MMNM_26
	21,666	snp	C	T	MMNM_33
R5-2	18,508	snp	T	C	MMNM_26
R5-3	18,508	snp	T	C	MMNM_26
	19,552	snp	C	T	MMNM_27
	19,645	snp	G	C	MMNM_27
R6-1	18,655	snp	C	T	MMNM_26
R6-2	15,895	snp	A	G	MMNM_23
	19,666	snp	T	C	MMNM_27
	41,938	snp	T	C	MMNM_56
R6-3	18,625	snp	C	T	MMNM_26
	19,645	snp	G	C	MMNM_27

^*a*^ REF refers to the nucleotide in the reference.

^*b*^ ALT The alternate nucleotide in the mutant.

^*c*^ The significant of the gray shading highlighted that the evolved phages were from the host AJ156.

At day 1, the plaques formed by phage MMNM(Ala_134_) on the permissive host *Klebsiella* B5055 were of uniform size and had a clear bulls-eye morphology (Fig. 3B). In an initial pilot experiment, 12 days of evolution on cultures in the presence of the semi-permissive host *Klebsiella* AJ174-2 was found to be sufficient to observe a range of different plaque size morphologies on the *Klebsiella* B5055 host (Fig. 3C; Fig. S2). For example, numerous smaller-sized plaques without the bulls-eye morphology were present (Fig. 3C, R5-3) as well as plaques that had turbid morphology (Fig. 3C, R5-2). No such variation in plaque morphology was observed in a control experiment run in the presence of a strictly non-permissive host, *Klebsiella* AJ303 (Fig. S2A). Thus, the presence of *Klebsiella* AJ174-2 is determining a selection for changes in plaque morphology, visualized on *Klebsiella* B5055.

Five of the evolved phages (E156-1 and E156-2, as well as R1-2, R1-3, and R6-2) were directly tested for binding to *Klebsiella* B5055 and *Klebsiella* AJ174-2 in phage adsorption assays (Fig. 3D). During the course of a co-incubation with the host strains *Klebsiella* B5055 or *Klebsiella* AJ174-2, samples were removed and tested for plaque-forming activity on *Klebsiella* B5055. Through the course of 20 min co-incubation, phages are seen to be lost from the sample co-incubated with *Klebsiella* B5055, consistent with a proportion of phage binding to the cell surfaces. A similar proportion of phage binding was observed in all cases shown (Fig. 3D) and in the other phages tested subsequently (Fig. S3). There was no measurable loss of phage due to co-incubation with the semi-permissive host *Klebsiella* AJ174-2.

### The presence of AJ174-2 selects for changes in the baseplate of phage MMNM(Ala_134_)

**Fig 4:**
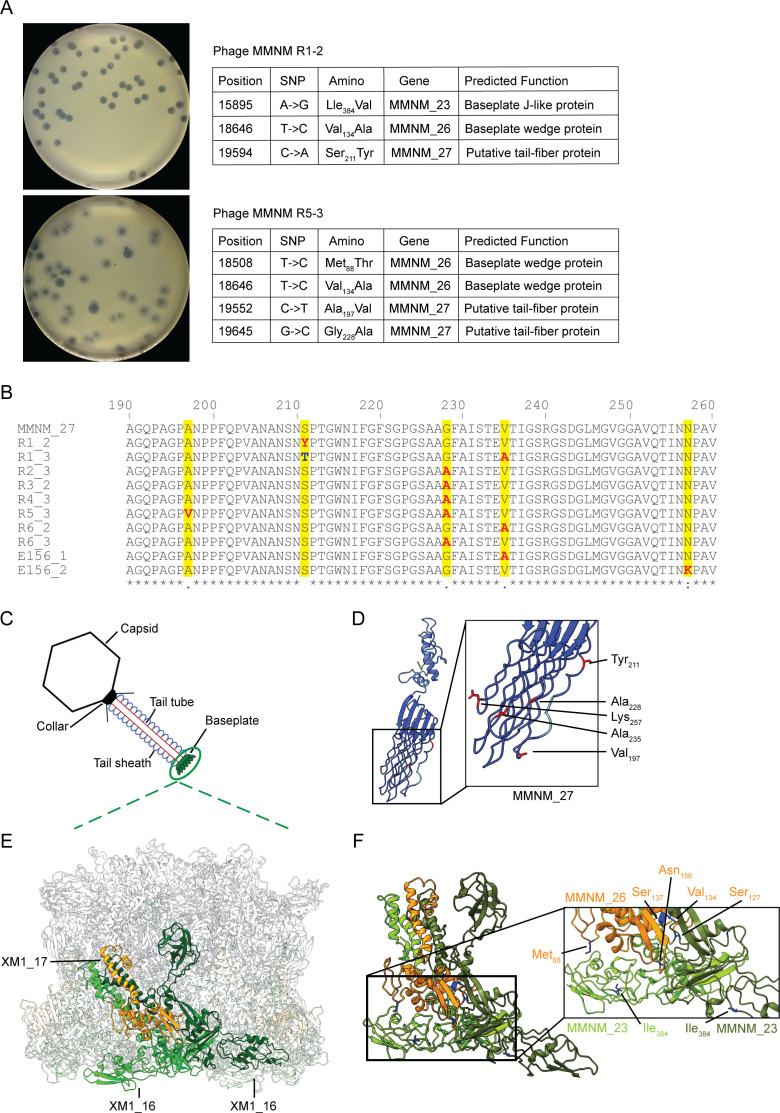
Mutations in the phage tail proteins selected in the presence of *Klebsiella* AJ174-2. (**A**) Data for purified phage MMNM R1-2 and phage MMNM R5-3, plated to show evolved plaque morphology and sequence summary of residues in the proteins, had been mutated. SNP indicates a single nucleotide polymorphism change from the ancestral MMNM genome to the evolved phage genome. (**B**) A segment of the multiple sequence alignment (see Fig. S4) showing the variant residues in each of the evolved phages, in this region of the tail-fiber protein MMNM_27. (**C**) Schematic representation of phage MMNM indicating the tail-fiber/baseplate region, corresponding to (**D**) the AlphaFold2 structure prediction for the putative tail-fiber protein MMNM_27, colored by predicted local distance difference test (pLDDT) confidence measure. Blue suggests a high-confidence prediction for a C-terminal β-sandwich domain, with long loops joining each of the β-strands. The prediction is of high confidence with the majority of the protein having >80% confidence score and the C-terminal domain being >90% confidence; some of the inter-strand loops score lower than the β-strands but rarely dip below 90% confidence (Fig. S4B). Indicated in the inset panel is a zoomed view of the inter-strand loops with the position (red) of residues mutated during the evolution of the phages. (**E**) Cryo-EM structure of the baseplate of phage XM1 (PDB:7KH1) highlighting the XM1_17 subunit (orange) and each of its neighboring XM1_16 subunits (light green and dark green). (**F**) The putative structures of MMNM_23 (green) and MMNM_26 (orange), predicted by fitting the MMNM sequences to the electron density map of baseplate homolog phage XM1 (50). The baseplate subunits are oriented such that the bottom of the graphic is the estimated contact point with the host during infection (50). Shown are the positions and residues mutated during the evolution of the phage.

Two of the evolved phages (designated E156-1 and E156-2) were isolated for genome sequence analysis (Table 2). In the subsequent larger-scale experiment for 12 days of evolution in the presence of *Klebsiella* B5055 and *Klebsiella* AJ174-2, a further 18 evolved phages were identified and isolated for genome sequence analysis (Fig. S2B). These short-term evolution experiments did not detect phages that could productively infect the new host, as there were no plaques formed when the evolved phage preparations were plated on lawns of *Klebsiella* AJ174-2. However, the phenotypic changes evident from the frequency of the smaller and turbid plaque morphologies (Fig. 4A) after the challenge with the potential new host were unexpected and were therefore analyzed by genome sequencing of the 20 individuals representing the evolved MMNM phages.

The SNPs identified across the 20 genomes were present in a limited set of open-reading frames (Table 2). The vast majority of the phenotypically variant phages selected for by competition on a non-permissive host had established variation in the base-plate structure and tail-fiber proteins of the virion (Fig. S2C). A single hit was seen for the MMNM_24 (depolymerase) in phage R3-2 (a single +, Table 1), but the deletion does not change the open-reading frame (see Materials and Methods). Instead, the plaque morphology phenotype changes seen in phage MMNM-R3-2 could be due to the mutations in the other tail-proteins MMNM_26 and MMNM_27.

### Structural evaluation of the minimalist phage

There is evidence suggesting that variation in the base-plate structure and tail-fiber proteins is causative for the plaque morphology differences. First, we note that a mutation in MMNM_26 was the only change in a protein that evolved in phages MMNM-R1-1, -R2-1, -R3-3, -R5-2, and -R6-1. Second, a mutation in MMNM_23 was the only change in the evolved phage MMNM-R2-2 (Table 2). Third, phage MMNM-R1-2 had evolved to yield smaller plaques (with mutations in tail proteins MMNM_23 and MMNM_27), and phage MMNM(Ala_134_)-R5-3 had evolved to yield turbid plaques with mutations in tail proteins MMNM_26 and MMNM_27 (Fig. 4A).

We note that in the case where mutations in non-virion proteins were recovered (MMNM_4, MMNM_6, MMNM_64, MMNM_33, and MMNM_56), they were recovered in phages that had evolved to also carry mutations in the tail proteins MMNM_23 and/or MMNM_26 and/or MMNM_27 (Table 2).

In the case of MMNM_27, the 11 mutations recovered (11 +, Table 1) are conservative substitutions. For example, hydroxylated residues at position 211 (T_211_ or Y_211_ in place of S_211_) or non-polar residues at position 197 (V_197_ in place of A_197_ and or A_235_ in place of V_235_). The variant residues mapped on a multiple sequence alignment showed no obvious pattern other than occurring in the C-terminal half of the protein sequence (Fig. 4B; Fig. S4A), and these were considered in the context of the tail-fiber/base-plate structure of the phage virion (Fig. 4C). An AlphaFold predicted three-dimensional model resolved a clear pattern wherein all of the mutations that evolved in MMNM_27 occurred in residues where the side-chains emanate from the long inter-strand loops. As a result, they are all displayed on the same surface relative to the plane of the β-sandwich domain in the predicted structure (Fig. 4D; Fig. S4B). These long inter-strand loops are also found in the tail-fiber protein gp24 (PDB:7YFW) in the related phage Pam3. This would be consistent with the annotation of the protein as a “tail-fiber,” and the HHpred prediction that MMNM_27 is related to the tail fiber, not the baseplate proteins, of phage Pam3.

In the case of the Baseplate wedge protein (MMNM_26) and Baseplate J-like protein (MMNM_23), the identity of the proteins is sufficiently high to homologous proteins in phage XM1 (50) that a structural model of the predicted interaction between MMNM_26 and MMNM_23 could be built from the corresponding subunits XM1_16 (Fig. 4E, green) and XM1_17 (Fig. 4E, orange) in the XM1 baseplate structure. A bottom-up view of the baseplate shows that these two protein subunits span from the outer face of the baseplate to the channel within the baseplate (Fig. S4C), and because of the sixfold symmetry of the base-plate, there are alternating XM_16 and XM_17 subunits around the baseplate perimeter. Displayed in the context of a comparative structural model, residues S_137_, V_134_, S_127_, and N_156_ of MMNM_26 are all located at what would be the interfacial surface between MMNM_26 and MMNM_23 (Fig. 4F) in the predicted structure of the phage baseplate. While mere speculation at this stage, the selection of these changes in the interfacial surfaces may impact the rate at which these subunits can be assembled into the baseplate in order to generate more phage progeny in the initial phase of replication before any phage defense system triggers the death of the bacterial population. However, the alternative is that the evolved variation in the baseplate is to better engage with a receptor variant in the semi-permissive host.

## DISCUSSION

The interaction between lytic phages, such as phage MMNM, and their bacterial host is purely antagonistic with selection for phages to kill bacteria in order to propagate (36). Lytic phages bind to surface receptors on a bacterial cell and then inject in their DNA into the cytoplasm in order that (i) it can be transcribed and translated to make new phage virion proteins, and (ii) the genome can be replicated for packaging into the new phage virions. The progeny phage then lyse the bacterium and hunt for new bacteria to engage *ad infinitum* (32, 51, 52). Permissive hosts, therefore, exert selection for the parental phage genotype for as long as it is the most competitive genotype in a population. However, we reasoned that a phage subpopulation that could at least minimally suppress the phage defense mechanism in *Klebsiella* AJ174-2 to allow for some phage progeny and no ongoing selection would provide an opportunity to isolate variants that are functional but not yet evolved to maximal efficiency. Our hypothesis was that phage MMNM(Ala_134_) would provide a snapshot of antagonistic co-evolution, given the abrupt, non-selective period of its interaction with the semi-permissive host *Klebsiella* AJ174-2.

Serendipitously, visualization of the minimalist phage virions also provided a unique opportunity to address how far into the periplasm a phage tail-tube can penetrate. According to the paradigm for phage infection, phages such as MMNM should contract after the tail-fibers encounter receptors on the host cell surface, wherein the phage tail structure contraction punctures the bacterial cell envelope and releases phage genomic DNA into the host cytoplasm (7, 53). By observing around a thousand phage MMNM profiles, we found enough virions contracted during sample handling that we could measure how much of the tail tube protrudes from the contracted sheath. The lack of additional tail-fiber proteins in the minimalist phage meant that the extruded inner tail-tube was readily visible for length measurement. The protruding 30 nm of tail tube would not be sufficient to reach to the cytoplasm of a host bacterium: even if the capsule (380 nm) and O-antigen (26 nm) layers (15) are ignored, the distance from the outside surface of the outer membrane to the inside surface of the inner membrane is 41 nm [(41); see Fig. S1]. There is a constructive debate in the field about phage penetration of the inner membrane, with cryo-electron tomography and other structural studies providing evidence of phages either assembling new tubular structures to help reach the cytoplasm or phage tail penetration somehow signaling for a massive movement of the inner membrane apposing to the outer membrane and thereby minimizing the distance to be traversed (7, 54–59). It remains to be investigated as to which additional features need to remodeled or reconstructed inside the host cell envelope in order that the genomic DNA can be translocated into the cytoplasm, and we suggest that, with fewer components to consider, the five baseplate proteins of the minimalist phage MMNM provide an excellent model for discovery of the relevant features that pave the way into the cytoplasm.

### Minimalist phage baseplates

The baseplate is a complex piece of molecular machinery and is situated in the capsid-distal end of the phage tail-tube, where it functions to enable the outer sheath to contract back from the inner tail-tube (60–67). The current model for the assembly of contractile phage virions sees the baseplate as being the earliest step in the assembly process (67). In the classic example of phage T4, the structure of the baseplate is known from electron microscopy of the virions and X-ray crystallography of the individual proteins (60, 68, 69). The radial symmetry is such that a “hub” formed of multiple protein subunits is situated at the end of the phage tail, around which radiates multiple wedge proteins. In contrast to this elaborate baseplate, two recent studies presented single-particle cryo-electron microscopy analysis of what can be considered minimalist contractile phages: Pam3 and XM1. Phage Pam3 was isolated from a freshwater lake in China where it infects a cyanobacterial host, *Pseudoanabaena mucicola* (70), and phage XM1 infects *Vibrio harveyi* and *Vibrio rotiferianus* in marine environments. Cryo-electron microscopy showed that phage Pam3 has 23 structural proteins, with only six of these proteins in the base-plate structure (67). Three proteins (gp18, gp19, and gp20) form the hub structure that includes a plug and tail-spike, two proteins (gp22 and gp23) form the base-plate wedges, and a single globular protein (gp24) forms the radiating “tail-fiber.” A similarly minimalist baseplate structure has been deposited for phage XM1 (Fig. 4E).

The morphology of phage Pam3 (67) and phage XM1 (50) represents models for the architecture of phage MMNM, both in terms of the smaller number of components predicted in the base-plate and in the non-fibrous, globular form of the tail-fiber protein. These phages have limited overall sequence similarity and belong to different genera (Fig. S5). However, the proteins in their respective baseplate structures have conserved structural features recognizable by AlphaFold. In the case of Pam3, the structure of the baseplate-associated tail-fiber (PDB:7YFW) is similar to the AlphaFold predicted structure of tail-fiber MMNM_27. From the cryo-electron microscopy of phage Pam3 and XM1, the base-plate wedge proteins and the tail-fiber exhibit the most solvent-exposed surface that would be available to adsorb to bacterial cell surfaces. The base-plate proteins (MMNM_23 and MMNM_26) and tail-fiber protein (MMNM_27) were the site of most mutations in the evolved forms of phage MMNM (Table 1), consistent with the base-plate and its associated fibers assisting initial adsorption to *Klebsiella* cell surfaces, prior to its function in driving contraction.

### Experimental evolution, natural selection, and host-range

In an evolutionary sense, the antagonistic relationship between phages and their host bacteria selects both for phage-resistant changes in the bacterial host and for changes in the phage proteins that may ultimately change the host-range (8, 9, 36, 52, 71). To adapt to evolutionary changes in a host or to diversify host-range, phage evolution would require either (i) major genetic events to swap components from one phage to another producing mosaicism, or (ii) multiple small mutations to be fixed into a sub-population (8, 36). An example of a major genetic event would be where the simultaneous infection of one bacterial cell by two or more phages enabled the exchange of genetic material and the provision of new depolymerases (27, 28), with such events thought to explain the genetic mosaicism observed in phylogenies drawn from phage genomes (29–31). Our focus has instead been on establishing systems where we might observe multiple small mutations providing the diversity on which co-evolution could act, and it was for this reason that the seemingly innocuous non-fatal impact of phage MMNM(Ala_134_) on *Klebsiella* strain in its K2 capsule-type host-range caught our attention. Subsequent analysis showed that this strain, *Klebsiella* AJ174-2, expresses little if any capsular polysaccharide and has a differential sensitivity to phages MMNM and MMNM(Ala_134_), and further mutations in the baseplate proteins of phage MMNM(Ala_134_) could be selected for by co-cultivation of *Klebsiella* AJ174-2 and a permissive host (B5055) to amplify these evolved phages.

We are left with a hypothesis that a host immunity system in *Klebsiella* AJ174-2 aborts infections effectively against phage MMNM, but less so against the evolved phages (8, 9, 72). We have not demonstrated that the classic *abi* mechanism is responsible for protecting *Klebsiella* AJ174-2 from phage MMNM. However, this or some other means of antiphage defense is consistent with the observations that (i) phage MMNM(Ala_134_) and its evolved forms generate a spot of inhibited growth in a lawn of *Klebsiella* AJ174-2 in spot-test assays, yet (ii) there was insufficient progeny phage generated in one-step growth curve assessments of *Klebsiella* AJ174-2, and (iii) there is a specific *Klebsiella* AJ174-2-dependent recovery of mutant phages as judged by differential phenotypes (plaque morphology) on the permissive host *Klebsiella* B5055 after evolution experiments. Potentially, the mutant phages assemble more rapidly and thus have a slightly increased success rate in infection so that more infection events occur across the population before the suppression of replication is triggered. The mechanism behind this is worthy of future investigation, but here, we have investigated the outcomes of the evolutionary process.

Phage MMNM showed itself to have a contractile tail, and the Baseplate J-like protein (MMNM_23) and Baseplate wedge protein (MMNM_26) would form key components of that structure (7, 60). The variant residue that distinguishes phage MMNM and phage MMNM(Ala_134_) virions is present in the baseplate wedge protein MMNM_26. In addition to this founding variation, 13 further mutations were identified in the phages that were evolved by co-incubation with its permissive *Klebsiella* host B5055 and the semi-permissive AJ174-2. No such variation evolved when a non-permissive *Klebsiella* AJ303 host was used.

### Phage tails can evolve incrementally

For *Klebsiella* and other encapsulated bacteria, many phages can use a two-step mechanism to recognize their host (16): the first step is to engage with the capsular polysaccharide through a receptor binding protein that binds (and can hydrolyze) the capsule, and the second step engaging a receptor in the outer membrane which is often a protein but can alternatively be the O-antigen of the lipopolysaccharide (LPS) (16, 73–75). The TraDIS screen presented here is consistent with phages derived from MMNM making some use of capsule K2 for binding but where LPS (type O1) is needed to function as the phage receptor.

In the short-term evolution experiments presented here, 31 of the 37 mutations recovered across the 20 evolved phages are predicted to be part of the baseplate. The other 6 out of 37 mutations were in either a predicted lytic transglycosylase (MMNM_19), a predicted coil-containing protein (MMNM_65), the hypothetical protein MMNM_04, or the protein MMNM_24 which was shown to function as a depolymerase active against K2 capsular polysaccharide and experimentally validated to be attached to the baseplate as well as the capsid-proximal end of the phage tail (Fig. 1J).

The majority of the mutations (31 of the 37) selected were in the putative baseplate J-like protein MMNM_23, baseplate wedge protein MMNM_26, and tail-fiber protein MMNM_27. The initial indications based on homology modeling is that these mutations might be preferentially found in interfacial surfaces between MMNM_23 and MMNM_26 in the baseplate or displayed on flexible loops emanating from the tail-fiber protein MMNM_27. The evolved phages carrying these mutations were recovered only in experiments where the non-permissive host *Klebsiella* AJ174-2 was present.

Whatever the infection scenario, the data presented are consistent with a conclusion that the baseplate structure of these minimalist phages plays a role in host-range specificity beyond its attributed function in phage tail contraction. It is also consistent with a phenotype of successful phage entry into the new *Klebsiella* AJ174-2 host where the replicative cycle is suppressed to allow but a few new phage to emerge for evolution to act on in small phenotypic changes (8). We suggest that the community module system employing the minimalist phage offers the opportunity to further explore how well single nucleotide variation could provide a driver to alter receptor-binding and other phenotypes of phages in ways that would be permissive to altered host interactions (4, 8, 33, 34).

## MATERIALS AND METHODS

### Bacterial strains

The *Klebsiella* strains assayed to establish host-range have been documented previously (16), and many are clinical isolates from the Alfred Hospital collection that have been characterized previously (76). Initial analysis of one of these isolates, *Klebsiella* AJ156, showed clearance zones for phage MMNM(Ala) but not phage MMNM. However, re-streaking of the *Klebsiella* AJ156 stock and colony morphology analysis showed that two strains were present in the original clinical isolate: one of K25 capsule type forming opaque colonies [AJ156 as per the original documentation (77)] and another with a K2 capsule type that formed more translucent colonies. We refer to this strain with the differential phage sensitivity as *Klebsiella* AJ174-2. Genomic DNA was prepared from each of the strains for DNA sequencing and the data deposited at NCBI with the following accession numbers: CP159309–CP159313 (*Klebsiella* AJ156) and CP159314–CP159316 (*Klebsiella* AJ174-2).

### Phage isolation and infection *of Klebsiella*

Water samples from the Merri Creek (Melbourne, Australia) were centrifuged at 10,000 × *g* for 10 min and filtered through a 0.45 µm cut-off filter. The filtered water samples (45 mL) were subsequently mixed with 5 mL of 10× concentrated Luria-Bertani (LB) media and 1 mL of a *Klebsiella* B5055 Δ*ompK36* overnight culture and grown for a further 16 h at 37°C. Cellular debris was removed by centrifugation at 10,000 × *g* for 10 min, and the resultant clarified supernatant was passed through a 0.45 µm filter. Phage infectivity via spot and plaque assays was subsequently measured according to a published procedure (35).

### Phage amplification, purification, and phenotyping

Phages were amplified and purified on caesium chloride (CsCl) gradients using procedures previously described (35), where phage MMNM was previously visualized, and one-step growth curve experiments were performed as previously described (78). Plaque morphology was visualized after plaque assays via liquid infections and top agar overlays (16). Infection plates were subsequently imaged using a Phenobooth (Singer Instruments) using the default camera settings. To assess host range, phages were incubated with *Klebsiella* spp. strains from a collection that has been previously described (16).

To assess phage virion morphology by electron microscopy, CsCl-purified phage samples were prepared as described previously (35), and immuno-gold labeling of phage virions was assessed as previously described (79) using antiserum raised to purified MMNM_24. Purified high-titer phage preparations (4 µL) were added to freshly glow-discharged CF200-Cu Carbon Support Film 200 Mesh Copper grids (ProSciTech) for 30 seconds. The sample was blotted from the grid using Whatman filter paper, and samples were subsequently stained with 4 µL of Nano W Methylamine Tungstate (Nanoprobes) for 30 seconds and blotted again. Grids were imaged using a Tecnai Spirit G2 transmission electron microscope (Tecnai).

### Anti-MMNM_24 antiserum production

The depolymerase MMNM_24 was purified as follows. The open-reading frame coding for MMNM_24 was cloned into the protein expression vector pPROEX htb (Thermo Fisher) following PCR amplification with the primers M1dpFNco (forwards: 5′-CTATCCCATGGCCATTATCAAACGTGCAGACC-3′) and M1dpRXho (reverse: 5′-ATACCCTCGAGCTATGTGAAATTGATGATGAAATTATCG-3′) from purified phage template. N-terminally his-tagged MMNM_24 was expressed in *E. coli* strain BL21 DE3 Star (Novagen), in Terrific broth (TB) media at 18°C overnight, with shaking, following induction with 0.2 mM IPTG (isopropyl β- d-1-thiogalactopyranoside). Cells were harvested by centrifugation, and the resultant cell pellet resuspended in lysis buffer [20 mM Tris pH 8.0, 400 mM NaCl, 0.5 mM MgCl_2_, 20 mM imidazole, and complete EDTA-free protease inhibitor (Roche)] and lysed in an Avestin Emulsiflex C3 cell press (three passes). Following centrifugation (30 min at 30,000 × *g*, 4°C), the clarified lysate was applied to a 5 mL HisTrap column (Cytiva) and eluted by application of a gradient of 20 mM Tris, 400 mM NaCl, and 1 M imidazole (16). The MMNM_24 containing fractions were pooled and further purified via size exclusion chromatography over a HiLoad 16/600 Superdex 200 pg (Cytiva) equilibrated in 25 mM Tris pH 8.0 and 150 mM NaCl.

Protein analysis routinely made use of SDS-PAGE as previously described (80). Crude and partially purified extracts as well as purified MMNM_24 were assessed by Coomassie-stained reducing SDS-PAGE. Purified MMNM_24 was submitted to the WEHI Antibody Production Facility (https://www.wehi.edu.au/) to generate a polyclonal anti-MMNM_24 antiserum.

### Capsule extraction, quantification, and Alcian blue staining

Capsular polysaccharide was purified from a 10 mL culture of *Klebsiella* B5055 grown in LB to an OD_600_ of ~0.5 prior to harvesting by centrifugation (6,000 × *g*, 10 min, at 4°C) exactly according to reference (16). The polysaccharide was resuspended in Tris-buffered saline (TBS). Ten microgram of purified polysaccharide was incubated with a defined amount (2.5 µg or 10 µg) of purified MMNM_24 protein or an equivalent volume of TBS for 1 h at 37°C. Briefly, the gel was washed in fixing buffer [25% (vol/vol) ethanol and 10% (vol/vol) acetic acid in Milli-Q water] three times at 50°C (10 min each wash), before staining with 0.125% (wt/vol) Alcian blue in fixing buffer (for 15 min at 50°C in the dark). The gel was destained with a fixing buffer at room temperature and visualized.

### Experimental evolution of MMNM(Ala_134_) phage

To establish a community module for experimental evolution, a two-host treatment in which a permissive host (*Klebsiella* B5055) and semi-permissive host (*Klebsiella* AJ174-2) or non-permissive host (*Klebsiella* AJ303) were prepared in a ratio of 1:1 with a total density of 10^8^ CFU/mL and used for phage MMNM(Ala_134_) infection. All host bacteria were freshly cultured from stocks prior to the next-day infection. Phage lysates were mixed with bacterial culture at a multiplicity of infection of 0.1, followed by an overnight incubation shaking at 37°C. Phage lysates were then harvested and used to infect cultures for the subsequent day of the evolution period. The overall process was repeated every day for 12 days. Initially, two selection (B5055/AJ174-2 and B5055/AJ303) pilot experiments were carried out. After that, the selection of two-host treatment (B5055/AJ174-2) with six replicates was conducted. All replicates of evolved phage lysate were subjected to spot assays and plaque assays in order to determine the capacity of the evolved phage to infect each of the hosts present in their treatment.

### Phage genomic DNA extraction, sequencing, and annotation

For phage MMNM(Ala_134_) and for the additional phages isolated after the evolution experiments, phage genomic DNA was isolated from 1.8 mL of phage working stock lysates (~10^10^ PFU/mL) as described in reference (16). The purity and concentration of the extracted DNA were measured using Nanodrop and Qubit double-stranded DNA BR assay kit (Thermo Scientific). Whole-genome sequencing was performed by Victorian Clinical Genetics Services, using Illumina NovaSeq 6000 platform with a paired-end run of 2 × 150 bp. Raw reads were quality-checked and assembled using Shovill (https://github.com/tseemann/shovill). Mosdepth (https://github.com/brentp/mosdepth) and nf-core/viralrecon (https://github.com/nf-core/viralrecon) pipeline were also used to analyze MMNM(Ala_134_) genome. Mutations of the evolved phages were identified using Snippy v4.6.0 mapping and variant calling pipeline (https://github.com/tseemann/snippy) and mapped against the annotated feature of reference genome MMNM (Accession No: MT894004.1). In all cases, the potential consequences to protein coding were evaluated by manual analysis. For example, manual evaluation of the single hit seen for the MMNM_24 (depolymerase) in phage R3-2 revealed that the mutation represented the deletion of “G” residue from a stretch of residues that contribute a “G” to the STOP codon “TAG.” Thus, in the sequence context of the MMNM_24 open-reading frame: ACA TA**G** GGG GGG GGT ATT, is converted to ACA TA**G** GGG GGG GTA TT, with no consequence to the open-reading frame that encodes MMNM_24.

### AlphaFold2 predictions of phage proteins

AlphaFold2 predictions were made using the ColabFold tool, version 1.5.2-patch (81), as updated on 12 June 2023, which relies on the MMseqs2 and AlphaFold2 systems in conjunction with Google Colaboratory to predict protein folding. Each query sequence was run as a monomer using the amino acid sequence. Settings were unchanged other than using the pdb100 template mode to detect potential templates already present. Predicted proteins were visualized using the ChimeraX software, version 1.5 (82).

### Electron density mapping for phage protein prediction

Homology modeling of the baseplate proteins MMNM_23 and MMNM_26 was done using the phage XM1 electron microscopy map [(50) Protein Databank (PDB) accession number: 7KH1]. XM1 was chosen based on high HHpred (83) similarity of MMNM_23 and MMNM_26. Initial fitting of MMNM_23 and MMNM_26 to XM1_16 and XM1_17, respectively, was done using Swiss PdbViewer version 4.1.0 (84) using the magic fit tool. The fitted protein was then screened with Coot version 0.9.8.7 (85) to remove erroneous residues. ChimeraX software, version 1.6.1 (82) with the ISOLDE version 1.6.0 (86) add-on was used to minimize energy levels against the XM1 electron microscopy map, as well as visualize the proteins and sites of mutation.

### TraDIS to identify essential host genes for phage infection

A high-density transposon mutant library of *Klebsiella* B5055 containing ∼200,000 unique insertion sites (45) was used to define mutants surviving treatment with phage MMNM(Ala_134_). Three cultures were grown in LB medium, each inoculated with 10^9^ bacterial cells from the transposon library stock and 10^10^ MMNM(Ala_134_) viral particles. Cultures were grown for a further 5 h, centrifuged (6,000 × *g*, 10 min, at 4°C), and the cell pellets were washed in 10 mM Tris. Genomic DNA was isolated from each cell pellet by phenol-chloroform extraction using 15 mL phase lock tubes (Qiagen). Two microgram of each gDNA preparation was used to prepare transposon-specific sequencing libraries using primer FS108 for specific amplification of transposon junctions as described previously (87). DNA libraries were sequenced using the Illumina MiSeq platform with primer FS107 as described previously (46).

TraDIS analysis to determine receptor identity was performed essentially as described previously (16). Reads from transposon-gDNA junctions were mapped to the B5055 genome (GenBank accession no. CP072200–CP072202) using the BioTraDIS pipeline with the parameters “-v smalt_r −1 t TAAGAGACAG -mm 1” and assigned to genomic features, with reads mapping to the 3′ 10% of the gene ignored. Comparisons between phage-treated and control samples were performed using the “TraDIS_comparison_positive_selection.R” script (https://github.com/francesca-short/tradis_scripts), which is based on the comparison script from the BioTraDIS toolkit but, in addition, reports the insertion index ratio between condition and control samples. Filtering based on gene-wise transposon mutant diversity (insertion index ratio) was necessary because, for many of the genes with increased read counts post-phage challenge, these reads mapped to just a single insertion site. These cases were presumed to result from rare secondary mutations unrelated to the transposon insertion, as suggested previously (88). Genes required for phage infection were defined as those with a log2 fold change of  >1, a *q* value of  < 0.01, and an insertion index ratio of ≥1 between the phage-treated and input samples.

## Data Availability

Raw sequencing data of evolved phages and TraDIS have been deposited in GenBank under the Bioproject accession number PRJNA1020759 and PRJNA1139334, respectively. The genome sequence of semi-permissive host Klebsiella AJ174-2 has been deposited with accession number CP159314–CP159316.

## SUPPLEMENTAL MATERIAL

Supplemental Figures mbio.02564-24-s0001.pdf Figures S1-S5.

Table S1 mbio.02564-24-s0002.pdf Host-range analyses.

Table S2 mbio.02564-24-s0003.xlsx Phage defense systems.

ASM does not own the copyrights to Supplemental Material that may be linked to, or accessed through, an article. The authors have granted ASM a non-exclusive, world-wide license to publish the Supplemental Material files. Please contact the corresponding author directly for reuse.
