# Supplementary material for: Genetic variation in individuals from a population of the minimalist bacteriophage Merri-merri-uth nyilam marra-natj driving evolution of the virus
Source: mBio. 2024 Oct 30;15(12):e02564-24. doi: 10.1128/mbio.02564-24 (PMC11633184; doi:10.1128/mbio.02564-24)
Supplement: Supplemental Figures — Figures S1-S5. [file mbio.02564-24-s0001.pdf]

A

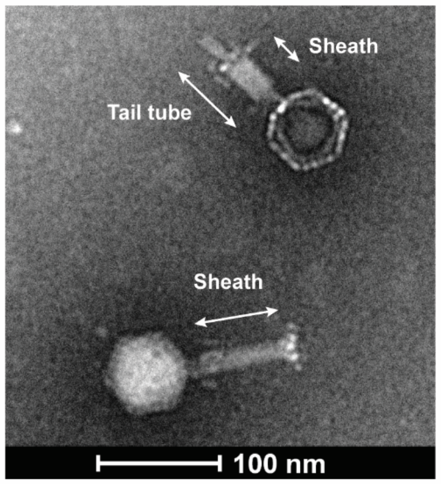

B

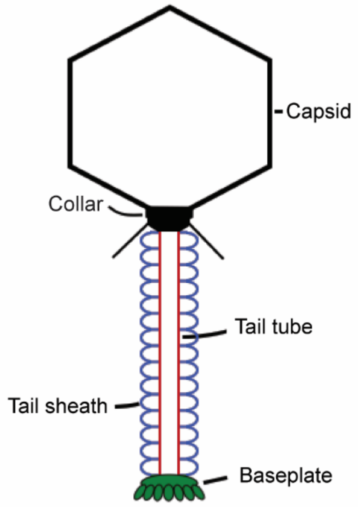

C

	Tail-tube	sheath
Contracted	85.17	49.02
	86.68	56.08
	84.32	54.91
	89.62	57.12
	79.66	51.38
	79.20	53.54
	81.78	55.69
Average	83.78	53.96

	sheath
Non-Contracted	90.40
	91.77
	91.19
	90.40
	91.19
	95.50
	90.21
	90.01
	90.60
	93.34
	88.83
	91.00
	90.40
	92.17
	92.17
	91.00
	90.60
	88.50
	92.36
	92.96
	93.34
	95.11
	92.55
	94.32
	93.74
Average	91.75

D

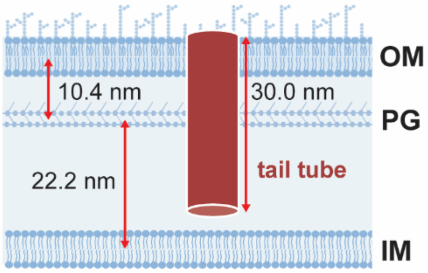

Figure S1. Phage MMNM is a contractile phage that could penetrate up to 30 nm into a host bacterium.

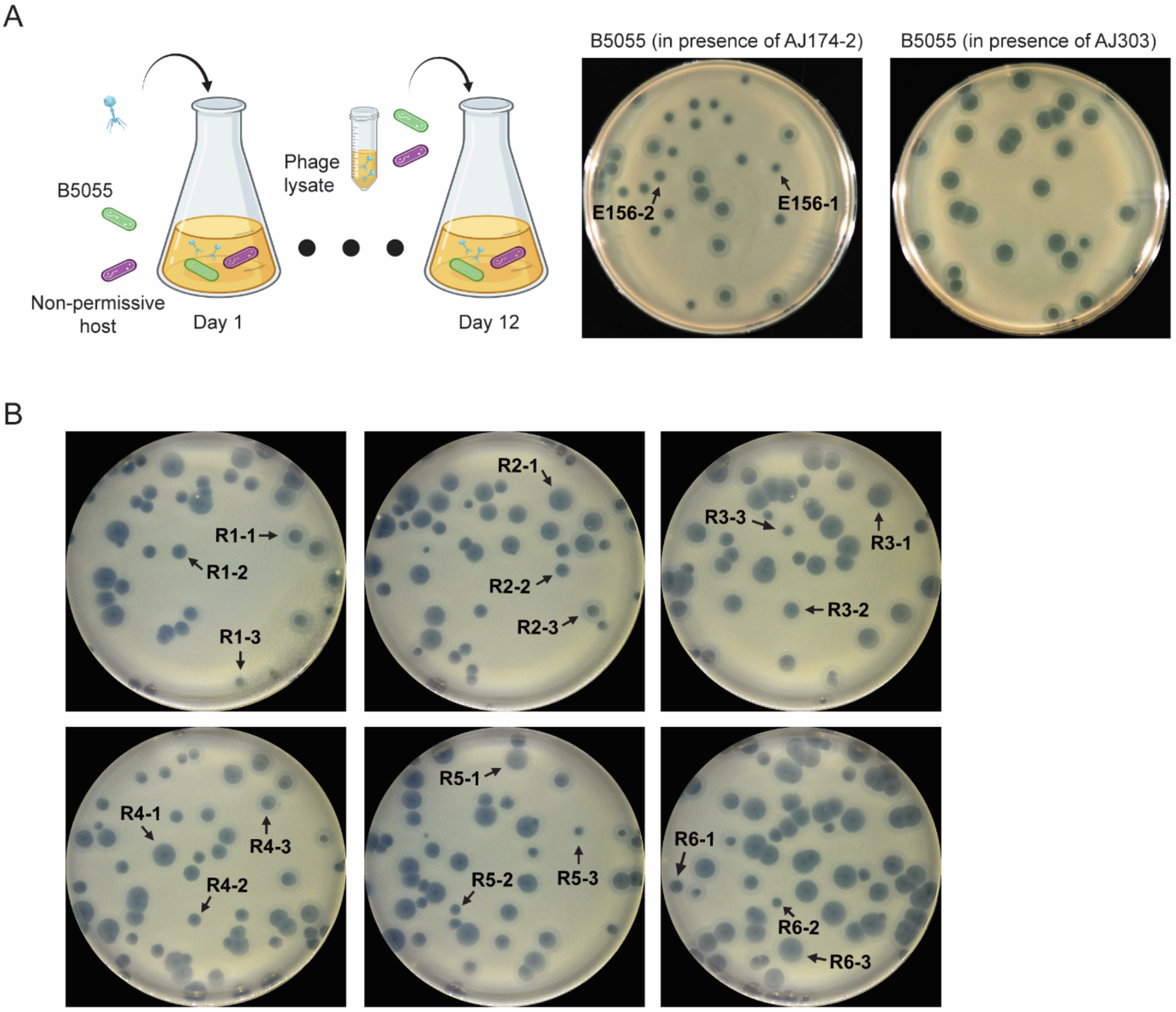

C

Protein	MMNM_23	MMNM_24	MMNM_26	MMNM_27
Phages with variant protein	E156-1 (His$_{384}$Arg)<br>E156-2 (His$_{384}$Arg)<br>R1-2 (Lle$_{384}$Val)<br>R1-3 (Lle$_{384}$Val)<br>R2-2 (Lle$_{384}$Val)<br>R4-2 (Lle$_{384}$Val)<br>R6-2 (His$_{384}$Arg)	R3-2 (Del Ag$_{669}$)	R1-1 (Ser$_{127}$Phe)<br>R2-1 (Met$_{88}$Thr)<br>R2-3 (Ser$_{127}$Phe)<br>R3-1 (Ser$_{127}$Phe)<br>R3-2 (Met$_{88}$Thr)<br>R3-3 (Asn$_{156}$Ser)<br>R4-1 (Met$_{88}$Thr)<br>R4-3 (Ser$_{127}$Phe)<br>R5-1 (Met$_{88}$Thr)<br>R5-2 (Met$_{88}$Thr)<br>R5-3 (Met$_{88}$Thr)<br>R6-1 (Ser$_{127}$Phe)<br>R6-3 (Ser$_{127}$Phe)	E156-1 (Val$_{235}$Ala)<br>E156-2 (Asn$_{257}$Lys)<br>R1-2 (Ser$_{211}$Tyr)<br>R1-3 (Val$_{235}$Ala)<br>R2-3 (Gly$_{228}$Ala)<br>R3-2 (Gly$_{228}$Ala)<br>R4-3 (Gly$_{228}$Ala)<br>R5-3 (Ala$_{197}$Val)<br>R5-3 (Gly$_{228}$Ala)<br>R6-2 (Val$_{235}$Ala)<br>R6-3 (Gly$_{228}$Ala)

Figure S2. Mutations in phage tail proteins selected in the presence of *Klebsiella* AJ174-2.

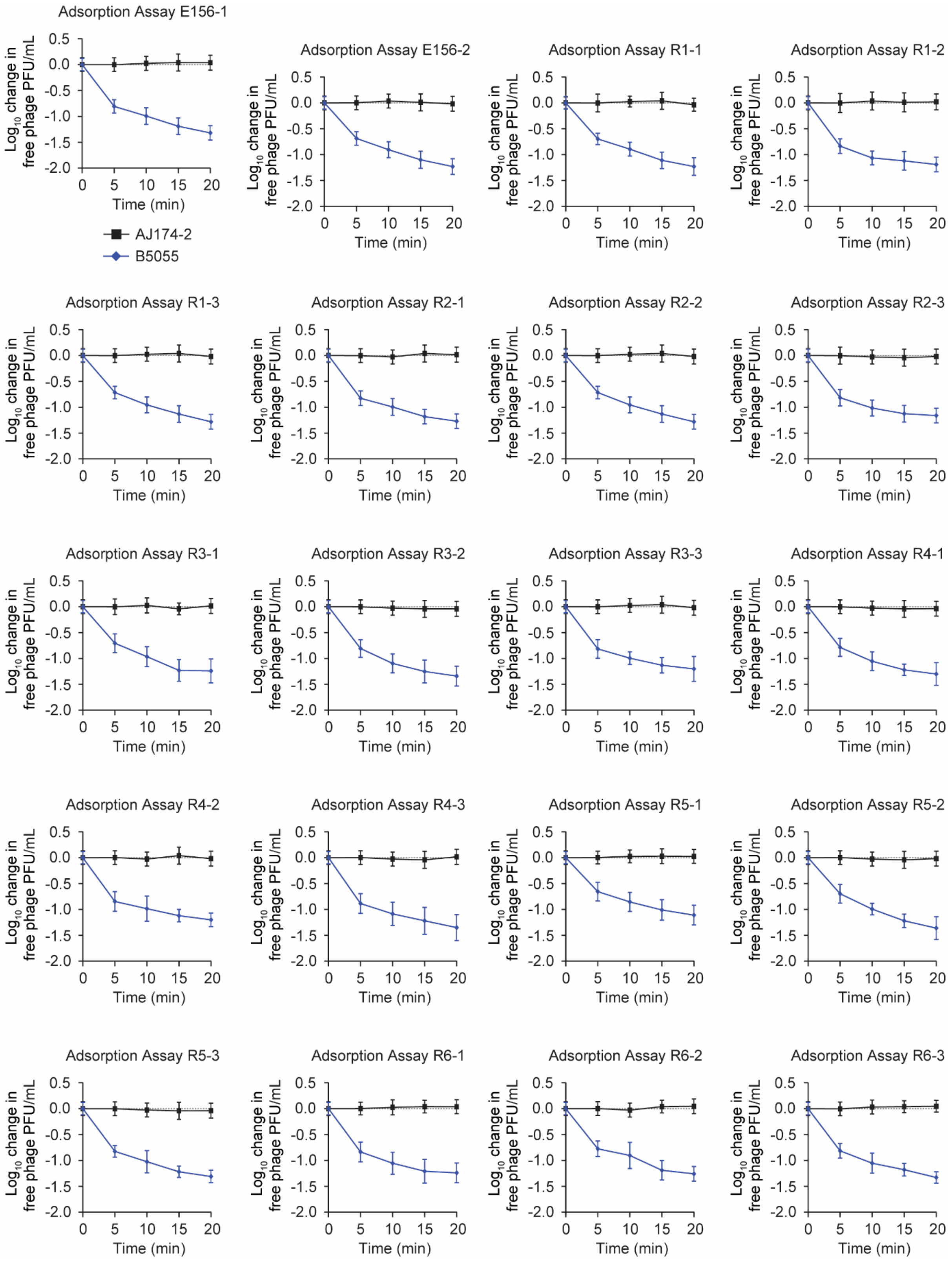

Figure S3. Adsorption assay of evolved phages on B5055 and AJ174-2.

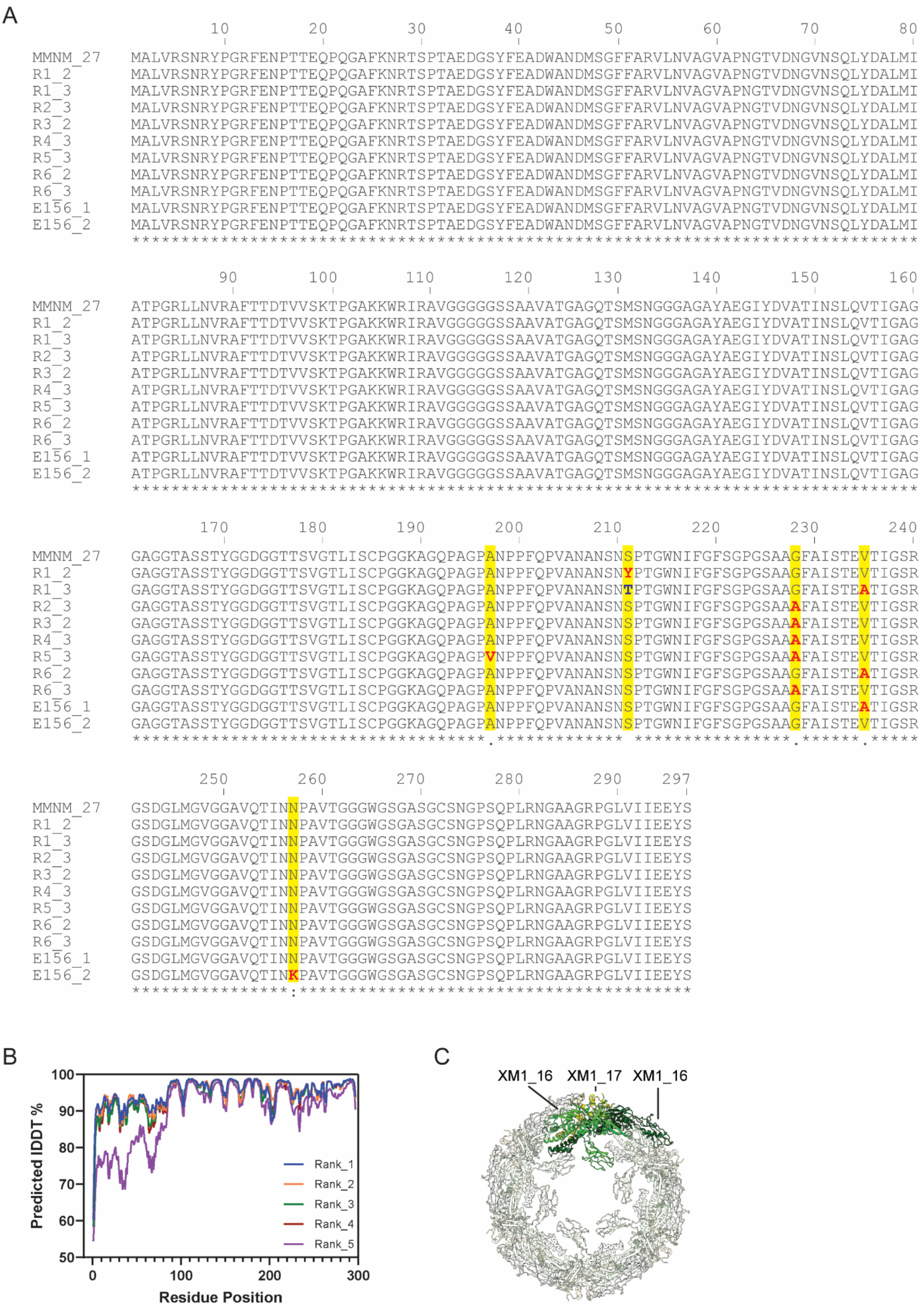

Figure S4. Sequence summary of the evolved changes in putative tail-fibre protein MMNM_27.

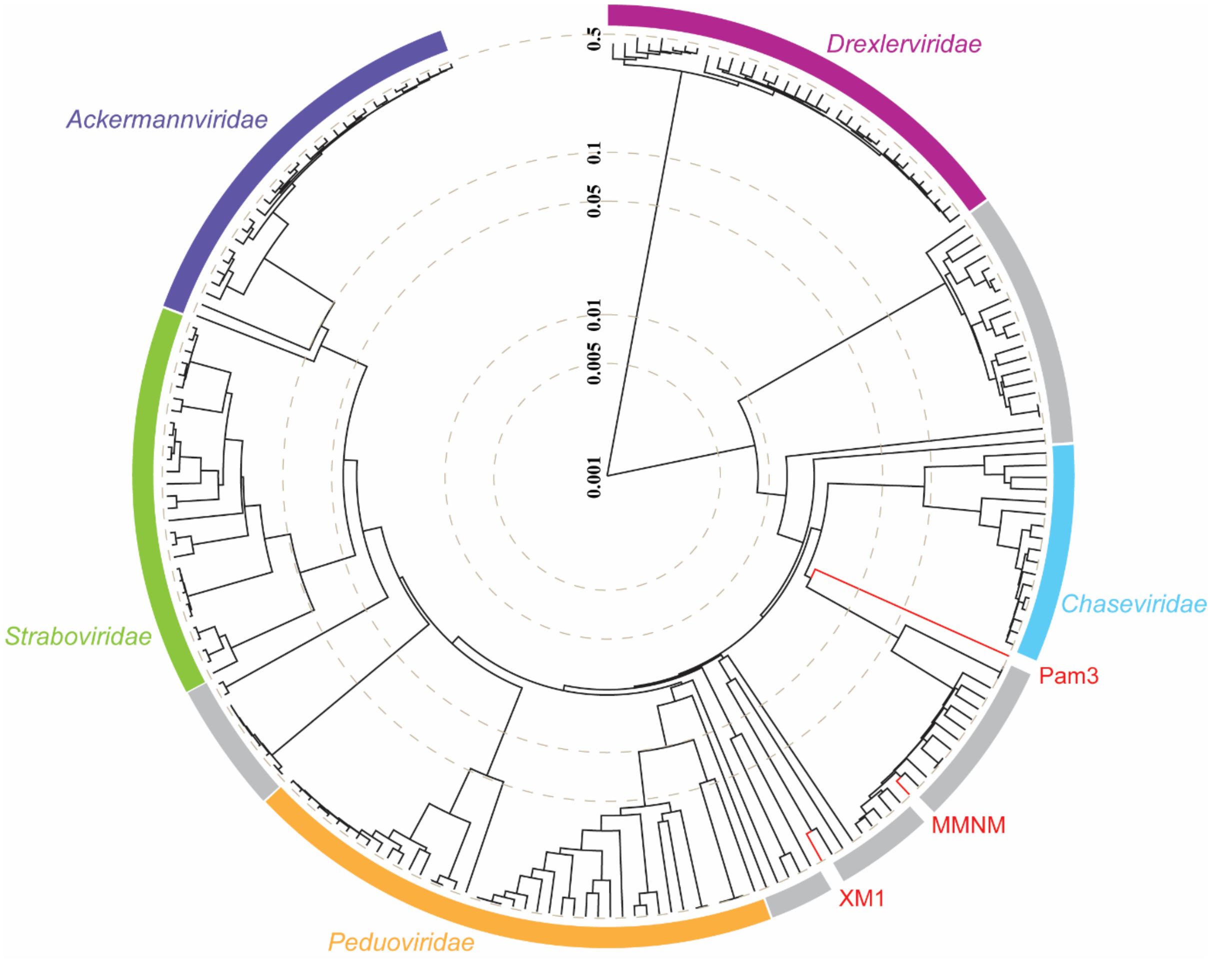

Figure S5. Relationship of MMNM phage to phage Pam3 and phage XM1.
