# Supplementary material for: Genetic variation in individuals from a population of the minimalist bacteriophage Merri-merri-uth nyilam marra-natj driving evolution of the virus
Source: mBio. 2024 Oct 30;15(12):e02564-24. doi: 10.1128/mbio.02564-24 (PMC11633184; doi:10.1128/mbio.02564-24)
Supplement: Table S1 — Host-range analyses. [file mbio.02564-24-s0002.pdf]

Supplementary Table S1. Host specificity for phage MMNM.
Strains used in this study: B5055, AJ174-2, AJ303 and AJ156 are highlighted.

Strain	Capsule-type	MMNM	MMNM (Ala_{134})
AJ033	K0	-	-
AJ196	K1	-	-
B5055	K2	+++	+++
AJ174-2	K2	-	*
AJ214	K3	-	-
AJ188	K5	-	-
AJ031	K6	-	-
AJ146	K9	-	-
AJ082	K11	-	-
AJ289	K16	-	-
AJ026	K17	-	-
AJ303	K21	-	-
AJ158	K22	-	-
AJ156	K25	-	-
AJ229	K26	-	-
AJ054	K27	-	-
AJ006	K28	-	-
AJ205	K30	-	-
AJ170	K38	-	-
AJ056	K49	-	-
MGH78578	K52	-	-
AJ218	K54	-	-
AJ135	K60	-	-
AJ292	K61	-	-
AJ049	K64	-	-
AJ027	K81	-	-

Note: +++ denotes clear lytic spot, * denotes turbid spot, and – denotes lack of phage infection as assessed by spot test assays.
